# How Could Artificial Intelligence Change the Doctor–Patient Relationship? A Medical Ethics Perspective

**DOI:** 10.3390/healthcare13182340

**Published:** 2025-09-17

**Authors:** Gianluca Montanari Vergallo, Laura Leondina Campanozzi, Matteo Gulino, Lorena Bassis, Pasquale Ricci, Simona Zaami, Susanna Marinelli, Vittoradolfo Tambone, Paola Frati

**Affiliations:** 1 Department of Anatomical, Histological, Medico-Legal and Orthopaedic Sciences, «Sapienza» University of Rome, 00185 Roma, Italy; gianluca.montanarivergallo@uniroma1.it (G.M.V.); simona.zaami@uniroma1.it (S.Z.); paola.frati@uniroma1.it (P.F.); 2 Research Unit of Bioethics and Humanities, Campus Bio-Medico University of Rome, 00128 Roma, Italy; l.campanozzi@unicampus.it (L.L.C.); v.tambone@unicampus.it (V.T.); 3 Department of Clinical Sciences and Translational Medicine, University of Rome Tor Vergata, 00133 Rome, Italy; matteo.gulino@uniroma2.it; 4 Department of Life and Health Sciences, “Link Campus University” of Rome, 00165 Roma, Italy; p.ricci@unilink.it; 5 School of Law, Polytechnic University of Marche, 60121 Ancona, Italy; s.marinelli@pm.univpm.it; 6 Operative Research Unit of Department of Clinical Affairs, Fondazione Policlinico Universitario Campus Bio-Medico, 00128 Roma, Italy

**Keywords:** artificial intelligence, doctor–patient relationship, medical ethics, informed consent, confidentiality, patient autonomy, medical professionalism

## Abstract

**Background:** This paper aims to outline an ethical overview of the potential challenges related to AI technologies in the doctor–patient relationship. **Methods:** This study is structured as a narrative review of the literature (2015–2025), based on searches conducted in the main scientific databases (PubMed, Scopus, Web of Science, Google Scholar), supplemented by official documents issued by the following international organizations: World Health Organization (WHO), United Nations Educational, Scientific and Cultural Organization (UNESCO), and the World Medical Association (WMA), as well as key regulatory frameworks of the European Union, China, and the United States. The selection included academic contributions, guidelines, and institutional reports relevant to the clinical applications of AI and their ethical and regulatory implications. Specifically, the analysis herein presented is grounded on four key aspects: the rationale for AI in patient care, informed consent about AI use, confidentiality, and the impact on the therapeutic alliance and medical professionalism. **Results and Conclusions:** Depending on their application, AI systems may offer benefits regarding the management of administrative burdens and in supporting clinical decisions. However, their applications in diagnostics, particularly in fields as radiology and dermatology, may also adversely impact the patient–doctor relationship and professional autonomy. Specifically, the implementation of these systems, including generative AI, may lead to increased healthcare costs and jeopardise the patient–doctor relationships by exposing patients’ confidentiality to new risks and reducing space for healthcare empathy and personalisation. The future of the medical profession and the doctor–patient relationship will largely depend on the types of artificial intelligence that are integrated into clinical practice and how effectively such additions are reconciled with core ethical values on which healthcare rests within our systems and societies.

## 1. Introduction

The core notion of artificial intelligence (AI) has been broadly debated since the 1950s; however, it was only at the turn of the millennium that AI saw an exponential growth due to the rise in and development of deep-learning algorithms, which brought about a significant breakthrough in the field [1].

While there is still no agreed-upon and universally acknowledged definition of Artificial Intelligence, a workable definition is as follows: ‘AI system’ means a machine-based system that is designed to operate with varying levels of autonomy and that may exhibit adaptiveness after deployment, and that, for explicit or implicit objectives, infers, from the input it receives, how to generate outputs such as predictions, content, recommendations, or decisions that can influence physical or virtual environments [2]. This definition offers us all a glimpse into the outstanding potential of AI, which is able to perform highly complex tasks based on data stores so large that they would be beyond the analytical power of any human being. Although the realization of this promising potential encompasses many sectors, healthcare is one of those where such an application is known to hold great promise. The new AI-based technologies have in fact proved particularly encouraging in this context, for instance, as a support for the healthcare professional in decision-making, through the use of empirical machine learning (ML) algorithms capable of learning from experience and new information and progressively improving their performance in the diagnostic, therapeutic, and prognostic phases in each individual case. That constitutes the very foundation of artificial intelligence (AI). Various types of machine learning exist, categorized by their data analysis methods and levels of dependency. In summary, machine learning (ML) is divided into three primary categories: ML that identifies patterns (unsupervised ML), ML that employs algorithms for classification and prediction based on prior examples (supervised ML), and ML that utilizes a reward and punishment system to develop a solution strategy aimed at addressing specific problems (reinforcement learning) [3]. The initial significant achievement of AI in the medical field began with the prediction of protein complexes in molecular medicine, which resulted in the discovery of new drug targets [4,5]. The extensive data contained within electronic medical records (EMRs) and hospital databases is ideally suited for AI analysis, providing valuable insights. Currently, EMRs are cumbersome and lack effective inter-record communication. Analysing this vast data reservoir is immensely beneficial. For instance, AI can identify individuals at risk for chronic diseases and expedite and enhance health system calculations of cost–benefit ratios, aiding in decision-making processes. Today, AI has been harnessed with growing regularity to analyse and learn to recognize patterns in image processing, generating significant interest in areas such as radiology, pathology, ophthalmology, and dermatology—collectively referred to as ‘vision-oriented specialties.’ The ability of AI to minimize common errors in routine clinical practice and make real-time predictions is remarkably important and effective.

Overall, AI has the potential to enhance healthcare practice by reducing both diagnostic and therapeutic uncertainty. It supports clinicians in the prevention, classification, and stratification of patients’ conditions; it predicts whether recovery requires specific treatment, and it interprets images and detects signals that are imperceptible to the human eye through data collection and analysis. Furthermore, AI contributes to understanding how and why diseases develop; it helps identify the most appropriate treatment options and assists physicians and healthcare professionals by providing access to updated and evidence-based guidelines, even in real time, both in the ward and during surgical procedures [6].

In order to better understand the opportunities and risks of these fascinating but controversial technologies, it is worth focusing on the use of AI in supporting diagnostics, particularly in radiology and dermatology [7,8]: automated systems have been designed that are able to read and recognize images through algorithms that have analysed an enormous amount of data, thus constituting valuable tools for identifying any suspicious lesions with speed, accuracy and precision. A good prediction allows for an accurate diagnosis. In fact, AI is a tool capable of implementing and developing those “predictive models” capable of detecting the onset of a disease early. In the specific case of radiology, in order to understand the use and added value of AI, an ad hoc study developed an algorithm capable of analysing and interpreting over one hundred thousand chest X-rays, which ultimately proved more accurate than professional assessments and opinions in detecting pneumonia [9].

AI applications have also been harnessed in various medical fields to assist with diagnoses. Such realms include, but are not limited to, cardiology [10,11,12], neurology [13,14], oncology [15,16], radiology [17], radiotherapy [18,19], ophthalmology [20], gastroenterology [21,22], gynaecologic oncology [23,24], breastology [25,26], haematology [27], and infectious diseases [28]. Furthermore, such models show significant promise in the diagnosis of autism [29,30,31]. This is particularly important due to the complex nature of autism, which involves varied aetiology, severity, co-morbid conditions, and challenges related to therapeutic management [32,33,34,35,36].

In view of the concrete and wide-ranging benefits that AI systems seem to foreshadow by improving healthcare quality, a critical evaluation of the potential disadvantages and consequences of these practices in routine clinical settings cannot be overlooked. Special attention should be paid to the fundamental cornerstone of the medical profession: the doctor–patient relationship.

Although it is still uncertain how and to what extent AI technologies may affect the doctor–patient relationship, there is no doubt that establishing and maintaining a good healthcare relationship is of utmost importance in the delivery of effective assistance, both in terms of care experience and clinical outcomes [37,38]. Such dynamics represent the very cornerstone of modern patient-centred medicine, wherein the healthcare relationship itself is regarded as essential, thus preceding and superseding any technological aid and serving as the quintessential avenue through which a substantial degree of medicine’s therapeutic capacity is harnessed [39].

Artificial intelligence systems hold considerable promise as valuable adjuncts across a wide range of clinical domains [40]. Yet, their deployment simultaneously gives rise to significant ethical concerns, which, if left unaddressed, risk undermining the trust, reciprocity, and overall quality of the physician–patient relationship (Table 1) [41,42,43,44,45,46,47]. Therefore, it is essential to strike a tenable balance between the utilization of AI-based technologies and maintaining a human and accountable relationship between medical professionals and their patients. In order to achieve such a balance, the fundamental principles of respect for patients and their rights must be upheld at all times, while also fulfilling the professional obligation to perform one’s duties to the best of our abilities.

This article aims to lay out an overview of the potential challenges related to AI technologies for the doctor–patient relationship. The scenario thus delineated will hopefully serve as a valuable framework for defining future research priorities and evidence-based practices for safeguarding and enhancing the therapeutic alliance. Any failure to acknowledge and respect well-balanced standards and boundaries can compromise the quality and effectiveness of care, thereby jeopardizing patient health and well-being.

## 2. Methods

The present contribution is a narrative review of the scientific literature and most significant international policy documents. This methodology was chosen in order to bring to the fore bioethical, medical, and regulatory perspectives, which can be challenging to fully outline through the strictly quantitative criteria of systematic reviews. The databases PubMed, Scopus, Web of Science, and Google Scholar were drawn upon, along with official documents, recommendations, and policy papers issued by the World Health Organization (WHO), the United Nations Educational, Scientific, and Cultural Organization (UNESCO), and the World Medical Association (WMA). Such institutions were selected by virtue of their authority and, consequently, for their capacity to influence future regulatory choices of individual states. To assess whether the recommendations of these international bodies have been incorporated within the European Union, China, and the United States, the principal regulatory frameworks of such nations (or supranational institutions such as the EU) were also accounted for. The EU, China, and the USA were selected as representative jurisdictions of the three principal world regions. The analysis included academic articles, guidelines, and institutional reports published between 2015 and 2025, focusing on clinical applications of AI as well as on ethical and legal aspects (informed consent, trust, autonomy, equity, dignity). This approach enabled the identification of four major thematic areas: grounds for employing AI technology for patients; communication about the use of AI to patients; confidentiality; and therapeutic alliance and healthcare professionalism. The authors excluded studies published in languages other than English or Italian, as well as those with a predominantly technical–engineering focus lacking ethical or legal analysis.

## 3. Results

### 3.1. Grounds for Employing AI Technology for Patients

On 23 November 2021, as part of its mandate, UNESCO adopted an important Recommendation addressing ethical issues related to AI, cutting across different domains of application. The document elaborates on a number of salient points that inform the investigation under consideration, and it is noteworthy for its explicit references to the necessity of investing in research that explores the impact of AI systems on human–human relationships, as well as of ensuring due attention is paid to the importance of the patient’s relationship with healthcare staff [48].

Among the ethical issues addressed, the document points out that the employment of AI systems must be supported by the following arguments: (a) the AI method selected should be appropriate, desirable, and proportionate to achieve a lawful aim; (b) the AI method selected should not violate or abuse human rights or infringe upon the foundational values; and (c) the chosen AI method should be suitable for the context and based on rigorous scientific principles [48].

At this point, it is highly advisable to adopt a cautious approach when deciding whether or not to implement an AI technology in a given context, taking into account respect for human dignity and prioritizing the patient’s well-being and health, and not a mere cost–benefit analysis based on an industry-like logic. It is worth taking into account, for example, an algorithm system designed to predict the life expectancy of seriously ill patients, identifying those who would not benefit from hospitalization and who should therefore stay at home. The rationale which the company adopted to delineate the decision-making process may be unknown, and yet such a system could risk violating the patient’s human dignity and autonomy by evaluating human beings and their life expectancy based predominantly on hidden financial interests [49].

Such aspects and complexities were addressed more extensively in the guidance document developed by the WHO on ‘Ethics and Governance of Artificial Intelligence for Health’ in June 2021. The premise of this report revolves around the need to move beyond an approach overly reliant on AI technologies as a panacea, since AI-based solutions are not immune to bias and error [50].

Specifically, the reliability of any AI tool is contingent upon the quality and quantity of the data employed in order to generate a clinical decision. If the data are not suitable for providing reliable indications with regard to the characteristics of the specific case, it is likely that the AI tool will make errors in diagnosis and therapy [50]. If there is strong clinical evidence that the AI system outperforms its human counterpart at specific tasks, then using the system to make precise, well-defined decisions may be fully justified. Some patients may experience preventable morbidity and death if judgments are left to humans when robots can complete them more quickly, precisely, and specifically. This is especially true if there is no chance of an offsetting benefit [50]. However, in the absence of such evidence, or if it is deemed to be inconclusive, relying on AI has the potential to adversely impact the quality and safety of patient care, thereby eroding the foundations of care relationships and the underlying trust.

Starting from St. Thomas Aquinas’s assertion that what is useful does not have in itself (i.e., insofar as it is useful) the reason for the good, but rather the reason as a means to the good (in the same way that a bitter medicine is useful insofar as it is conducive to recovery), it cannot therefore be argued that what is useful is, in and of itself, necessarily good. Rather, what is good is also truly useful [51]. This should lead us to reflect on the need for an adequate assessment approach to AI technologies, not so much in terms of a profitable strategy for the pursuit of specific interests in a given situation, but as an essential element of the general conditions for effective professional practice [52].

Accordingly, creating durable and privacy-preserving data access mechanisms that promote better training and validation of AI models using high-quality data will make AI much safer [48].

There is, therefore, broad agreement in the literature on the need to prevent artificial intelligence tools from being designed or programmed in ways that could infringe patients’ rights to health, self-determination, and confidentiality by prioritizing financial interests. From this perspective, AI should not alter the physician–patient relationship, which has been traditionally oriented toward safeguarding the patient under the physician’s responsibility.

### 3.2. Communication About the Use of AI to Patients

It is doubtful that hospitals or healthcare providers will disclose to patients that AI was utilized in a decision-making process to support, validate, or even replace a physician. There is no precedent for obtaining patients’ permission before using technology for therapeutic or diagnostic purposes. However, the foundation of informed consent and broader public confidence in healthcare may be called into question by the use of AI in medicine and the omission of its use. This problem arises from the question of whether the application of AI in decision-making and clinical treatment could compromise any of the rationales for obtaining informed consent, including protection, autonomy, averting abusive practices, trust, self-ownership, non-dominance, and personal integrity. Doctors should inform patients about the use of AI in an open and straightforward manner and in a timely fashion [53].

Continuing with the aforementioned example, if the AI system were configured to suggest a care pathway based on data concerning hospitalization expenses, and the physician accepted its predictions regarding the allocation of life opportunities without critical evaluation, the decision-making process would be adversely impacted, leading to inequitable treatment of patients overall [49]. While the patient ultimately holds the authority in the decision-making process, the way in which the available options are presented may influence their decisions, carrying significant implications for their autonomy and self-determination in healthcare choices.

Physicians have to make every effort to clarify to their patients the rationale behind the use of AI, its mechanisms, and its explainability, i.e., the degree to which humans can grasp the reasons behind a model’s decisions or predictions, which is instrumental in making the “black box” of complex AI systems more transparent and understandable, and in enabling users to comprehend why a given model arrived at a particular outcome. Healthcare professionals should therefore thoroughly expound upon the types of data that are gathered and relied on and how such data are utilized and shared with outside parties and elaborate on the security measures in place to uphold patient privacy. Physicians ought to be open and honest about any biases, privacy issues, or data breaches that may arise from the use of AI technology. The use of AI in healthcare and health science, including clinical trials and hospital practices, can only be successful in the long run by ensuring transparency, explainability, and intelligibility. All such components are functional in that they represent grounds for building trust, which is essential to advance an effective use of AI in medicine [50] (p. 26). Moreover, it is the explainability of an artificial intelligence decision that enables physicians to assess its accuracy and to use it as a “second opinion.” Consequently, explainability is essential if AI tools are to function as decision-support systems rather than substitutes for the physician’s decision-making role [54]. What is at stake in this context is not only professional ethics and ethical tenability, but the more deep-seated aspects underpinning the very essence of care, which cannot be reduced to a mere technical–scientific interaction with the inevitably depersonalizing overtones.

In circumstances where decisions are influenced by or based on AI algorithms, thorough information must be provided to patients, particularly in cases where such decisions may impact their safety or human rights. In such instances, individuals should also be entitled to request clarification from the relevant AI entity or public sector institution in charge of overseeing such techniques and their implementation.

Furthermore, it is crucial that individuals have the capacity to comprehend the rationales supporting every decision that has the potential to affect their rights and self-determination. In addition, they should be allowed to submit comments to a designated employee of a public or private sector organization who will be tasked with the examination and potential amendment of the decision. In instances where a good or service is delivered directly to customers or with the assistance of AI systems, it is incumbent upon AI actors to ensure that users are promptly and appropriately notified [48].

Therefore, it is essential to inform patients they are interacting with an AI system, since that can foster a trust-based relationship with healthcare professionals and the very institution of informed consent, and prevent the onset of novel and more insidious forms of paternalism in healthcare, which may be rooted in the unsupported claim that ‘computers know best’ [55].

Thus, the use of artificial intelligence in clinical practice appears to complicate the physician–patient relationship, as it requires the physician to inform patients about the adoption of such tools and, where possible, to explain their functioning and degree of reliability. Consequently, the time devoted to the human dimension of the therapeutic encounter should, in principle, increase.

### 3.3. Confidentiality

One of the most important values in preserving the human right to privacy in the relationship between doctors and patients is confidentiality. Its importance in the medical field has been acknowledged since the time of Hippocrates.

Safeguarding data is tantamount to safeguarding trust. When patients suspect that their health information could be circulated beyond the strict purposes of care, they are likely to withhold relevant details. Such withholding not only undermines the therapeutic alliance but also diminishes the physician’s ability to provide accurate diagnoses and effective treatment, thereby jeopardizing the overall quality of care [56].

Concerning the great advancement of AI systems in the healthcare sector, the recent Report commissioned by the Steering Committee for Human Rights in the field of Biomedicine and Health of the Council of Europe, in December 2021, has highlighted how unfettered innovation can pose a major threat on this front [57] (p. 55).

Specifically, when AI systems are developed, used, and relied upon with increasing regularity in healthcare, there may be a larger requirement to generate or choose high-quality real-world patient datasets for system testing and training purposes. In this regard, there are two ways that innovation could compromise confidentiality and privacy, as also pointed out by several studies. First, there is the granting of third-party access to (de-identified) patient data and electronic health records to test and build AI systems. Second, there exists a concern that physicians might be inclined to request additional tests and analyses for the purpose of training or assessing AI systems, rather than for their clinical benefit. This issue is especially pertinent as it subjects patients to unnecessary risks associated with data leaks or other breaches of confidentiality, alongside escalating healthcare expenses. The right to privacy may be infringed upon by any data generation that possesses dubious therapeutic value or is evidently driven solely by its usefulness for the testing or development of AI systems.

The utilization of patient health records for the purposes of testing and training AI systems should, at the very least, adhere to sufficient deidentification and privacy-enhancing protocols. One such option is differential privacy, which involves the addition of noise to prevent the identification of specific individuals in the dataset [57,58,59].

Similar concerns have also been expressed in the WHO guidance document, which recommends establishing clear and more comprehensive data management plans, stressing that confidentiality obligations alone may not be effective enough to protect data used for AI health technologies [50].

Given the potential need for medical monitoring, the United Nations Educational, Scientific, and Cultural Organization (UNESCO) has urged Member States to take special care when regulating prediction, detection, and treatment solutions for healthcare in AI applications, in addition to ensuring compliance with all applicable national and international data protection requirements, upholding privacy safeguards, and establishing effective mechanisms to ensure that individuals whose personal data is being analysed are informed about its use and grant consent thereof. Furthermore, this process should never hinder individual access to healthcare [48].

Confidentiality is not only a legal obligation, but an ethical and philosophical core value as well, which is part and parcel of the very nature of healthcare. Accordingly, AI in medicine must never forgo rigorous respect for confidentiality, in order to prevent the patient from losing control over their information, trust in the healthcare professionals, and ultimately, the perception of being treated as a human being and not as a mere statistic. Non-compliance with the value of confidentiality can potentially bring about a sense of alienation in patients, who may come to perceive themselves as being treated as mere ‘assets’ within a system that no longer acknowledges their individuality, but rather views them exclusively as a repository or source of health data.

Therefore, even in matters of confidentiality, artificial intelligence tools may pose significant risks. In this regard, and regardless of whether specific national regulations exist, healthcare professionals must adhere to their duty of confidentiality and uphold the primacy of patient welfare, ensuring that the protection of sensitive health information takes precedence over the commercial interests of AI developers and producers.

### 3.4. Therapeutic Alliance and Healthcare Professionalism

Numerous contemporary studies corroborate the findings of the Council of Europe Report, expressing concern that an excessive reliance on artificial intelligence by medical professionals—wherein they delegate total control of patient care to the technology—may result in an inappropriate dependence on such technology. This over-reliance could potentially lead to a decline in the skills of medical practitioners (de-skilling), ultimately diminishing their diagnostic sensitivity [57,60,61,62].

Shared decision-making (SDM) is genuinely achieved when both physicians and patients are able to contribute to the final choice (physicians by bringing medical expertise and empathy, and patients by providing information, values, and personal preferences) even when artificial intelligence is involved in the process [63]. The exclusion of physicians and patients from the decision-making process risks undermining physicians’ professional autonomy, whereby the extensive implementation of technological solutions can lead to the risk of a loss of control and exclusion from the dialogue, which is arguably essential for clinical care and shared decision-making, between healthcare professionals and patients [64,65,66,67]. Furthermore, if AI technology ultimately lessens the scope of communication and interaction between doctors and their patients, it can constrain the former’s ability to provide the patient with the most effective therapeutic interventions and thus compromise the principles of general supportive care, which include the benefits of human interaction when the patients are often most vulnerable [68]. AI therefore has the potential to further dehumanize medical practice and result in a return to paternalism, but this time with AI applying the rules instead of doctors [68,69]. For example, automated triage systems have expanded access to healthcare, particularly for underserved populations and communities with limited resources [70,71]. However, technological mediation carries the risk of diminishing the immediacy of human contact, thereby limiting the capacity to perceive non-verbal cues, emotions, and implicit needs and potentially weakening the therapeutic alliance as well as eroding the relational trust that underpins the physician–patient relationship [72,73]. Bioethical scholarship emphasizes that human dignity cannot be reduced to mere technical parameters [74,75]. The World Medical Association likewise underscores the primacy of the physician–patient relationship in the development and implementation of artificial intelligence systems in healthcare [76].

Furthermore, patients may turn to AI software for healthcare guidance, as AI-generated medical opinions become more widely available and supposedly trustworthy. Thus, the emergence of AI-generated medical opinions will probably cause a further change in the relationship between physicians, who have always held all the knowledge and experience, and patients or family members, who can now access increasingly sophisticated and accurate AI-generated opinions through large language model (LLM) chatbots like Google’s Bard or OpenAI’s GPT-4. Glass AI is a consumer technology designed specifically for the medical field. It is based on the GPT-4 algorithm and allows users to enter a clinical scenario to generate a differential diagnosis or clinical management plan. As such technologies become more widely available and popular, patients are likely to bring certain expectations to clinical examinations [77].

On the other hand, generative AI tools are increasingly employed to draft medical reports, synthesize clinical documentation, and render medical language more comprehensible to patients. In doing so, they enable physicians to perform their tasks more efficiently and, consequently, free up additional time to listen to patients, fully assess their needs, and set forth treatment alternatives and predicted outcomes, as AI helps with data retrieval and interpretation. Namely, physicians could spend more time with patients in contact, rather than concerning themselves with time-consuming bureaucratic duties and “paperwork” relative to electronic health records, which is known to create dissatisfaction and exhaustion [50,69,78,79,80,81,82].

In addition to the potential for time savings through the transcription and documentation processes, technology may also contribute to a reduction in physician burnout and to a deeper connection with the humanistic aspect of medicine. By enabling timely access to patient information in a comprehensible format, this approach can also contribute to the democratization of patient care [83].

AI technologies can facilitate contact and communication, thereby strengthening the doctor–patient bond, rather than disrupting it. For instance, they can analyse various treatment options so that the doctor can discuss them with the patient by disclosing benefits as well as risks [50]. They can also personalize information and make it more accessible to patients, in some cases more effectively than commonly used clinical guidelines [84].

Scientists are pushing the boundaries even further. In the future, tools already tested successfully in the commercial sector—capable of recognizing customers’ emotions through facial expressions as well as patterns of speech and writing—may find application in healthcare, with the aim of delivering increasingly personalized services [85]. Assistance in understanding the patient’s emotional state can be highly valuable for promptly addressing feelings of dissatisfaction which, if left unchecked, may undermine the relationship of trust. Nevertheless, it would be a serious mistake to delegate the human dimension of care to machines, even to the most advanced ones. Indeed, AI cannot provide the human contact, which is often seen as a way to show interest and empathy and even improve physicians’ diagnostic skills [86,87].

The potential inversion of the means–ends ratio with regard to the dimension of technique in medicine could result in a negative shift in the doctor–patient relationship. If the doctor comes to be viewed as a mere processor of data, rather than a provider of care, and the patient is reduced to a piece of statistics, rather than a person, the medical decision ultimately risks losing its dialogical value, because AI offers optimal solutions based on big data, thereby reducing the margin for personalization. It is imperative to acknowledge the distinction between physicians and technicians and to emphasize that such figures will never be interchangeable. This distinction is further compounded by the imperative that physicians should not be subject to the directives of robots or AI technologies. The doctor–patient relationship and alliance should be strengthened, not weakened; yet, as the literature indicates, AI entails not only benefits but also risks across every dimension of the physician–patient relationship (Table 2).

## 4. Discussion

The potential for improvement through the implementation of AI in healthcare is undeniable. For instance, telemedicine-based monitoring has shown a high degree of reliability and significant benefits for patients with serious conditions of diverse nature [89,90,91]. Furthermore, tools that automatically fill in electronic medical records, while doctors interact with patients, can improve the efficiency and effectiveness of medical consultations. Concerns have been raised regarding the utilization of AI tools capable of diagnosing and prescribing treatments, as such technologies have the potential to replace medical practitioners [92,93]. Additionally, with the inherently error-prone nature of AI, a consensus has been reached among prominent organizations such as UNESCO, the WHO and the CDBIO, as well as evidence-based research [94], that physicians should always retain their obligation to supervise the proper functioning of AI and control over the decision-making process [48,50,57]. Therefore, it would be wrong to simply assume that AI frees up doctors’ time, while it is reasonable to conclude that a more critical approach is needed in the implementation of these technologies within healthcare, with more targeted investment in critical thinking and reasoning [95]. In addition, in light of the unique complexities that AI will pose for each medical field where it is applied, the necessity to further customize AI risk assessment for particular medical fields has also been emphasized. Risk assessment should be tailored and customized to match the distinctive features of specific domains, as the clinical, social, and ethical risks and constraints vary across fields such as radiology, surgery, genomics, mental health, child health, and home care. For instance, in the area of radiology, several leading radiological associations from Europe and North America (including the American College of Radiology, European Society of Radiology, Radiological Society of North America, Society for Imaging Informatics in Medicine, European Society of Medical Imaging Informatics, Canadian Association of Radiologists, and American Association of Physicists in Medicine) collaborated to issue a statement [96] addressing the ethical dilemmas associated with the use of AI in radiology. All such societies have pointed out that the radiology scientific community should immediately start framing codes of ethics and practice for AI that encourage any application benefiting patients and the greater good. Similar efforts ought to be carried out and constantly updated as the technologies further evolve by other scientific societies in order to prioritize the ethical and equitable implementation and application.

In order to verify the validity of any AI-based decision, clinicians must undertake an evaluation of the same instrumental tests and clinical data that have previously been analysed by the artificial intelligence. That is to say, in order to check the correctness of the diagnosis and therapy, or any other contribution offered by the AI, the clinician must also make the diagnosis and decide on the therapy.

The use of generative AI for diagnosis and therapeutic decisions seems to further complicate medical practice and student training, as medical curricula should place greater importance on AI literacy to enhance patients’ understanding of the tools used by doctors, thereby fostering a trust-based relationship [69]. In addition to the usual information in the relationship with the patient, the physician will also have to explain how the chosen AI tool works, how reliable it is, and the logic and motives behind its use, also in relation to the protection of the health data involved. This additional informative work is a duty and a very complex task, which in turn needs to be balanced by other AI tools that carry out bureaucratic and repetitive activities.

The process of doctor–patient communication is more than the mere conveying of information; rather, it is a relational and interpretative endeavour that is built on trust, empathy, and mutual understanding. The integration of AI has the potential to affect such a delicate balance in several ways, leading to increased opacity and asymmetry in communication, especially if not all participants can access the same information and have the same level of understanding [97]. This may result in patient distrust, because of the absence or dearth of intelligible explanations, potentially undermining the sense of autonomy and even reducing their adherence to treatment cycles. Such concerns are indicative of the perspectives laid out by German philosopher Hans Jonas, who has cautioned against the potential unintended consequences of advanced technologies, positing that such innovations may paradoxically lead to higher uncertainty. Consequently, Jonas points to the need for an expansion of responsibility and awareness [98].

The aforementioned recommendations issued by WHO, UNESCO, and WMA, despite their authority, have not been uniformly implemented at the global level, as reflected in Table 3.

In Europe, the General Data Protection Regulation (GDPR) requires that data collection be limited to what is strictly necessary (data minimization), that it be used only in proportion to the stated purposes (proportionality), and that it be processed within a framework of responsibility and traceability (accountability), which entails obligations to document how and why such data are used. With specific regard to artificial intelligence, Article 22 of the GDPR establishes the right not to be subject to decisions based solely on automated processing [99].

Moreover, European Regulation 2024/1689 (the AI Act), Articles 14–15, classifies health-related systems as “high-risk,” thereby imposing requirements of technical robustness, transparency, explainability (the capacity to provide understandable reasons for algorithmic decisions), and human oversight, precisely to ensure that both patients and physicians retain ultimate control over algorithm-driven decisions [100].

Equally significant is Regulation 2017/745, Annex I, Section 23, which requires manufacturers of medical software to provide clear instructions to healthcare professionals, thereby enabling them to supply patients with adequate information regarding the artificial intelligence tools employed [101].

A completely different approach has been adopted by China. The Personal Information Protection Law, under Article 13, regulates the use of sensitive personal data, including health data, establishing consent as its legal basis. However, the law does not lay out exactly what kind of information must be provided to the patient: it is not mandatory to explain in detail how the algorithm functions; a general notice on the use of technological tools in the processing of personal data is considered sufficient. Moreover, Article 13 states that consent is not required in various circumstances, including when the use of personal data is necessary for the fulfilment of state duties or obligations, to respond to public health emergencies, or to safeguard the life, health, or property security of a natural person [102].

The 2022 Guidelines of the National Medical Products Administration (NMPA) on AI-based medical devices address technical requirements, clinical validation, and data quality. The document focuses primarily on the relationship between the manufacturer and the public authority, rather than on patient–physician communication [103]. Indeed, the NMPA regulations require that the limitations and performance of the algorithm be made known to physicians, who are however not bound to communicate this information to patients in a clear and comprehensible manner. Moreover, currently in place regulatory frameworks do not codify any provision upholding the patient’s right to challenge an algorithmic decision, to refuse its use in therapeutic settings, or to request further clarification. Consequently, the aforementioned guidelines do not establish binding transparency obligations toward patients comparable to those set forth in the European AI Act [104].

An intermediate model is represented by the United States, where no federal regulatory framework exists, unlike in Europe and China. In the United States as well, the applicable regulations stipulate that artificial intelligence may support—but never replace—the physician’s decision-making role [105].

However, patients in the United States are not granted the right to demand human review of an algorithmic decision, as is provided under the European AI Act. This does not preclude, on the one hand, the patient’s ability to refuse a treatment proposed on the basis of an algorithm, and, on the other hand, the possibility that, where a diagnosis or therapy has been determined through artificial intelligence, withholding such information from the patient may constitute a breach of the physician’s duty of disclosure and thus give rise to liability for damages [106].

According to the American Medical Association (one of the most prominent standard-bearers in clinical practice), the obligation to inform patients about the use of artificial intelligence is directly proportional to the level of risk that such technology may pose to patient safety [107]. Some scholars have pointed out that, in addition to the risk of harm, a further criterion should guide the choice between merely informing patients about the use of artificial intelligence tools, seeking their explicit consent, or omitting both information and consent: namely, the extent to which patients are able to exercise their decision-making power. At the heart of thoroughly informed consent lies counselling, which is affected by the underlying and novel distinctive traits that such innovative practices entail. That additional layer of complexity is therefore hardly surprising and common to other revolutionary techniques and approaches in medical care, e.g., epigenetics, gene editing, and personalized/precision medicine as a whole [108,109,110,111,112,113], all realms that are bound to be affected by AI, machine learning, and big data processing as well [109,111,112,113]. Counselling is therefore to be intended as an organic and multi-layered process, rather than a mere set of consultations, to make the patient fully aware and capable of understanding and assessing risks, benefits, and the unique repercussions of their choices on their lives. When both the risk of harm and the patient’s capacity to assert their will are high, obtaining consent is highly advisable. Conversely, when the risk of harm is low and there is no realistic prospect that disclosure would affect the patient’s choice, the physician is not required to provide such information [114].

**Table 3:** Regulatory comparison between the European Union, China, and the United States.

Dimension	European Union	China	United States
Main Regulatory Sources	- GDPR 2016/679 [99] - AI Act 2024/1689 [100] - Regulation 2017/745 [101]	- Personal Information Protection Law (PIPL, 2021) [102] - NMPA Guidelines (2022) [103]	- FDA, Clinical Decision Support Guidance (2022) [105] - FDA, AI/ML SaMD Action Plan (2021) [106] - Absence of a unified federal framework
AI–Physician Relationship	AI is not a substitute for human healthcare professionals, but it is to be viewed as an auxiliary tool	AI is not a substitute for human healthcare professionals but rather serves as an auxiliary tool	AI cannot replace human healthcare professionals but serves as an auxiliary tool
Transparency toward Patients	- Obligation to inform patients when AI affects diagnosis or therapy - Explainability - Right to be informed of limitations and margins of error	- No obligation to disclose the use of AI or to explain the functioning of the algorithm	- AMA (2024) [107]: disclosure proportional to the level of risk of harm
Right to Contest Algorithmic Decisions	- Right to human intervention and to refuse automated decisions (Art. 22 GDPR)	- Not provided	- Not recognized as a right visàvis the physician, although the latter may be held liable for failing to disclose the use of AI
Conception of the Physician–Patient Relationship	- Safeguardoriented model: primacy of patient autonomy	- Collectivist model: trust placed in the physician/institution; limited informed consent - Centrality of state and technical control	- Intermediate model: autonomy safeguarded through informed consent and physician’s legal responsibility

## 5. Conclusions

Future changes in the medical profession and in the doctor–patient relationship will depend on what types of artificial intelligence make their way into clinical practice. As mentioned earlier, if AI replaces doctors in bureaucratic and repetitive tasks, they will have more time for their patients and for the proper management of AI systems. If, instead, in addition to bureaucratic duties, doctors are required to understand how the AI generated a diagnosis or therapy in order to verify its soundness, the doctor–patient relationship could be negatively affected.

Alternatively, they could come to rely completely upon AI. However, in this scenario, doctors would lose their professional skills and autonomy. This concern is clearly expressed by the WHO, who state that “[i]f introduction of AI is not effectively managed, physicians could become dissatisfied and even leave medical practice” [50] (p. 61).

Certainly, as technology evolves, it is safe to say that AI and machine learning are still very far from thoroughly fulfilling their potential, whose boundaries are not even definable right now. In fact, as research in quantum computing advances, it leads to the emergence of quantum machine learning, which represents the intersection of quantum computing and artificial intelligence. This confluence promises to solve computational problems that are currently beyond the reach of even the most powerful “conventional” systems available today. The potential of this development is enormous and is likely to pave the way for increasingly powerful and far-reaching applications [115,116]. It is therefore impossible at the moment to conduct a remotely conclusive risk–benefit analysis or produce a definitive evaluation of what issues and quandaries might be on the horizon.

However, based on the analysis herein outlined, such changes will arguably have a positive impact, provided that the medical profession should maintain its current conception of artificial intelligence as a valuable asset in daily clinical practice, overseen by qualified and adequately trained healthcare professionals. This approach should be deemed essential to ensure the preservation of the doctor–patient relationship. The role of AI in healthcare should never be viewed as a surrogate or replacement for medical professionals. Instead, it should be considered a powerful means to the ultimate end of enhancing diagnostic accuracy, optimizing clinical processes, and freeing up time for what is indeed irreplaceable and non-negotiable: the human relationship with the patient. As Hans Jonas reminds us, technology must be responsible, and its application in medicine has to reinforce, rather than undermine, the ethics of care [98].

## Figures and Tables

**Table 1:** Main applications of artificial intelligence in healthcare and their impact on the therapeutic relationship.

AI Application in Healthcare	Opportunities	Main Ethical Concerns
Assisted Diagnostics (radiology, dermatology, digital pathology)	Increased diagnostic accuracy; reduction of errors; faster diagnosis	Shift of clinical authority from practitioner to algorithm; AI-related errors; de-skilling; challenges to informed consent; opacity of the decision-making process
Predictive Medicine and Big Data Analytics	Personalized prevention; improvement of care pathways	Excessive patient profiling; risk of stigmatization; data confidentiality issues
Generative AI (reports, clinical documentation, communication)	Faster, more timely drafting of reports and records, resulting in higher efficiency	Risk of errors or misleading information; loss of confidentiality
Telemedicine and Automated Triage	Broader and faster access to care; remote monitoring	Reduction of human interaction; exclusion of less digitally literate patients; risk of over-reliance on automated systems
Clinical Decision Support Systems (CDSSs)	Greater therapeutic precision; lower prescribing errors; fostering tailored, personalized forms of treatment	Shift of clinical authority from practitioner to algorithm; AI-related errors; de-skilling; challenges to informed consent; opacity of the decision-making process
Surgical and Assistive Robotics	Higher surgical precision; reduced invasivity; support in daily care activities	Physical and emotional distancing from patients; high costs and inequitable access; de-skilling

**Table 2:** How AI integration impacts different layers of the doctor–patient relationship.

Dimension	Potential Benefits	Ethical Risks and Critical Issues
Quality of Care	Greater diagnostic and therapeutic accuracy; personalization of treatments; predictive medicine	Possible errors due to data bias; opacity of AI systems; AI-related errors; professional de-skilling; programming choices that prioritize interests other than those of patients
Accessibility	Shorter waiting times; improved continuity of care; possibility of remote follow-up	Exclusion of less digitally literate patients; inequities in access due to economic or technological barriers
Physician Autonomy	Decision-making support; reduction in bureaucratic workload; more time for patient interaction	Professional de-skilling; shift of decision-making authority from clinician to AI system
Informed Consent and Trust	More time available to inform patients	Increased complexity of the information process; opacity of AI systems; loss of trust in physicians
Equity	Potential reduction in disparities through standardization	Limited usefulness of AI for minority groups, as algorithms are trained on datasets that do not adequately represent them [88]
Data Confidentiality	Automatic anonymization	Use of data without consent; breaches of anonymity

## Abbreviations

The following abbreviations are used in this manuscript:

AI	artificial intelligence
WHO	World Health Organization
WMA	World Medical Association
UNESCO	United Nations Educational, Social, and Cultural Organization
CDBIO	Council of Europe’s Steering Committee for Human Rights in the fields of Biomedicine and Health

## References

[1] Kaul V., Enslin S., Gross S.A. (2020). History of artificial intelligence in medicine. Gastrointest. Endosc..

[2] The European Parliament and the Council of the European Union (2024). Regulation (EU) 2024/1689 of the European Parliament and of the Council of 13 June 2024. Off. J. Eur. Union.

[3] Mahesh B. (2020). Machine Learning Algorithms—A Review. Int. J. Sci. Res..

[4] Hamet P., Tremblay J. (2017). Artificial Intelligence in Medicine. Metabolism.

[5] Kulkarni S., Seneviratne N., Baig M.S., Khan A.H.A. (2020). Artificial Intelligence in Medicine: Where Are We Now?. Acad. Radiol..

[6] Italian National Bioethics Committee and Italian National Committee for Biosafety, Biotechnologies and Life Sciences Joint Opinion on Artificial Intelligence and Medicine: Ethical Aspects. https://bioetica.governo.it/en/opinions/joint-opinions-icbicbbsl/artificial-intelligence-and-medicine-some-ethical-aspects/.

[7] Naylor C.D. (2018). On the prospects for a (deep) learning health care system. JAMA.

[8] Xiang F., Li Z., Jiang S., Li C., Li S., Gao T., He K., Chen J., Zhang J., Zhang J. (2025). Multimodal Masked Autoencoder Based on Adaptive Masking for Vitiligo Stage Classification. J. Imaging Inform. Med..

[9] Wang X., Peng Y., Lu L., Lu Z., Bagheri M., Summers R.M., Lu L., Wang X., Carneiro G., Yang L. (2019). Chest X-ray: Hospital-scale chest X-ray database and benchmarks on weakly supervised classification and localization of common thorax diseases. Deep Learning and Convolutional Neural Networks for Medical Imaging and Clinical Informatics.

[10] Vidal-Perez R., Vazquez-Rodriguez J.M. (2023). Role of artificial intelligence in cardiology. World J. Cardiol..

[11] Itchhaporia D. (2022). Artificial intelligence in cardiology. Trends Cardiovasc. Med..

[12] Sangaiah A.K., Arumugam M., Bian G.-B. (2020). An intelligent learning approach for improving ECG signal classification and arrhythmia analysis. Artif. Intell. Med..

[13] Hillis J.M., Bizzo B.C. (2022). Use of Artificial Intelligence in Clinical Neurology. Semin. Neurol..

[14] Cerasa A., Crowe B. (2024). Generative artificial intelligence in neurology: Opportunities and risks. Eur. J. Neurol..

[15] Bhinder B., Gilvary C., Madhukar N.S., Elemento O. (2021). Artificial Intelligence in Cancer Research and Precision Medicine. Cancer Discov..

[16] Chen Z.H., Lin L., Wu C.F., Li C.F., Xu R.H., Sun Y. (2021). Artificial intelligence for assisting cancer diagnosis and treatment in the era of precision medicine. Cancer Commun..

[17] Hosny A., Parmar C., Quackenbush J., Schwartz L.H., Aerts H.J.W.L. (2018). Artificial intelligence in radiology. Nat. Rev. Cancer.

[18] Li G., Wu X., Ma X. (2022). Artificial intelligence in radiotherapy. Semin. Cancer Biol..

[19] Siddique S., Chow J.C.L. (2020). Artificial intelligence in radiotherapy. Rep. Pract. Oncol. Radiother..

[20] Rottier J.B. (2018). Artificial intelligence: Reinforcing the place of humans in our healthcare system. Rev. Prat..

[21] Uche-Anya E., Anyane-Yeboa A., Berzin T.M., Ghassemi M., May F.P. (2022). Artificial intelligence in gastroenterology and hepatology: How to advance clinical practice while ensuring health equity. Gut.

[22] Kröner P.T., Engels M.M., Glicksberg B.S., Johnson K.W., Mzaik O., van Hooft J.E., Wallace M.B., El-Serag H.B., Krittanawong C. (2021). Artificial intelligence in gastroenterology: A state-of-the-art review. World J. Gastroenterol..

[23] Akazawa M., Hashimoto K. (2021). Artificial intelligence in gynecologic cancers: Current status and future challenges—A systematic review. Artif. Intell. Med..

[24] Jiang Y., Wang C., Zhou S. (2023). Artificial intelligence-based risk stratification, accurate diagnosis and treatment prediction in gynecologic oncology. Semin. Cancer Biol..

[25] Mundinger A., Mundinger C. (2024). Artificial Intelligence in Senology—Where Do We Stand and What Are the Future Horizons?. Eur. J. Breast Health.

[26] Bassi E., Russo A., Oliboni E., Zamboni F., De Santis C., Mansueto G., Montemezzi S., Foti G. (2024). The role of an artificial intelligence software in clinical senology: A mammography multi-reader study. Radiol. Med..

[27] Radakovich N. (2020). Artificial Intelligence in Hematology: Current Challenges and Opportunities. Curr. Hematol. Malig. Rep..

[28] Peiffer-Smadja N. (2020). Machine learning for clinical decision support in infectious diseases: A narrative review of current applications. Clin. Microbiol. Infect..

[29] Valizadeh A., Moassefi M., Nakhostin-Ansari A., Heidari Some’eh S., Hosseini-Asl H., Saghab Torbati M., Aghajani R., Maleki Ghorbani Z., Menbari-Oskouie I., Aghajani F. (2023). Automated diagnosis of autism with artificial intelligence: State of the art. Rev. Neurosci..

[30] Dufour M.M., Lanovaz M.J., Cardinal P. (2020). Artificial intelligence for the measurement of vocal stereotypy. J. Exp. Anal. Behav..

[31] Liu R., Salisbury J.P., Vahabzadeh A., Sahin N.T. (2017). Feasibility of an Autism-Focused Augmented Reality Smartglasses System for Social Communication and Behavioral Coaching. Front. Pediatr..

[32] Ferrara R., Iovino L., Di Renzo M., Ricci P. (2022). Babies under 1 year with atypical development: Perspectives for preventive individuation and treatment. Front. Psychol..

[33] Ferrara R., Ricci P., Damato F.M., Iovino L., Ricci L., Cicinelli G., Simeoli R., Keller R. (2023). Pregnancy in autistic women and social medical considerations: Scoping review and meta-synthesis. Front. Psychiatry.

[34] Ferrara R., Damato F., Iovino L., Marti F., Latina R., Colombi C., Ricci P. (2024). ESDM intervention in severe preschool autism: An Italian Case report, psychological and social medicine reflections. Ital. J. Pediatr..

[35] Ferrara R., Ansermet F., Massoni F., Petrone L., Onofri E., Ricci P., Archer T., Ricci S. (2016). Autism Spectrum Disorder and intact executive functioning. Clin. Ter..

[36] Troili G.M., Businaro R., Massoni F., Ricci L., Petrone L., Ricci P., Ricci S. (2013). Investigation on a group of autism children: Risk factors and medical social considerations. Clin. Ter..

[37] Nagy M., Sisk B. (2020). How will artificial intelligence affect patient-clinician relationships?. AMA J. Ethics.

[38] Wu Q., Jin Z., Wang P. (2022). The relationship between the physician-patient relationship, physician empathy, and patient trust. J. Gen. Intern. Med..

[39] Ardenghi S., Russo S., Rampoldi G., Bani M., Strepparava M.G. (2024). Medical students’ attitude toward patient-centeredness: A longitudinal study. Patient Educ. Couns..

[40] Poalelungi D.G., Musat C.L., Fulga A., Neagu M., Neagu A.I., Piraianu A.I., Fulga I. (2023). Advancing Patient Care: How Artificial Intelligence Is Transforming Healthcare. J. Pers. Med..

[41] Akingbola A., Adeleke O., Idris A., Adewole O., Adegbesan A. (2024). Artificial intelligence and the dehumanization of patient care. J. Med. Surg. Public Health.

[42] Mennella C., Maniscalco U., De Pietro G., Esposito M. (2024). Ethical and regulatory challenges of AI technologies in healthcare: A narrative review. Heliyon.

[43] Dankwa-Mullan I. (2024). Health Equity and Ethical Considerations in Using Artificial Intelligence in Public Health and Medicine. Prev. Cronic Dis..

[44] Ratti E., Morrison M. (2025). Ethical and social considerations of applying artificial intelligence in healthcare—A two-pronged scoping review. BMC Med. Ethics.

[45] Elendu C., Amaechi D.C., Elendu T.C., Jingwa K.A., Okoye O.K., Okah M.J., Ladele J.A., Farah A.H., Alimi H.A. (2023). Ethical implications of AI and robotics in healthcare: A review. Medicine.

[46] Chinta S.V., Wang Z., Palikhe A., Zhang X., Kashif A., Smith M.A., Liu J., Zhang W. (2025). AI-driven healthcare: A review on ensuring fairness and mitigating bias. PLoS Digit. Health.

[47] Weiner E.B., Dankwa-Mullan I., Nelson W.A., Hassanpour S. (2025). Ethical challenges and evolving strategies in the integration of artificial intelligence into clinical practice. PLoS Digit. Health.

[48] UNESCO Recommendation on the Ethics of Artificial Intelligence. Paris, France, 23 November 2021. https://www.unesco.org/en/articles/recommendation-ethics-artificial-intelligence.

[49] Woopen C., Paglia V., Pegoraro R. (2019). Ethical principles and democratic prerequisites for shaping robotics and artificial intelligence. Humans, Machines and Health: Proceedings of the XXV General Assembly of Members: Vatican City, Vatican, 25–27 February 2019.

[50] World Health Organization (WHO) (2021). Ethics and Governance of Artificial Intelligence for Health.

[51] Aquinas T. (1981). Summa Theologica, Fathers of the English Dominican Province, Trans.

[52] Lysdahl K.B., Oortwijn W., van der Wilt G.J., Refolo P., Sacchini D., Mozygemba K., Gerhardus A., Brereton L., Hofmann B. (2016). Ethical analysis in HTA of complex health interventions. BMC Med. Ethics.

[53] Prakash S., Balaji J.N., Joshi A., Surapaneni K.M. (2022). Ethical Conundrums in the Application of Artificial Intelligence (AI) in Healthcare—A Scoping Review of Reviews. J. Pers. Med..

[54] Hildt E. (2025). What Is the Role of Explainability in Medical Artificial Intelligence? A Case-Based Approach. Bioengineering.

[55] McDougall R.J. (2019). Computer knows best? The need for value-flexibility in medical AI. J. Med. Ethics.

[56] Nicholas N., Sotiris S. (2010). Understanding confidentiality and the law on access to medical records. Obstet. Gynaecol. Reprod. Med..

[57] Mittelstadt B. (2021). The Impact of Artificial Intelligence on the Doctor-Patient Relationship.

[58] Dwork C., Bugliesi M., Preneel B., Sasson V., Wegener I. (2006). Differential privacy. Automata, Languages and Programming.

[59] Ohm P. (2010). Broken promises of privacy: Responding to the surprising failure of anonymization. UCLA Law Rev..

[60] Chen Y., Stavropoulou C., Narasinkan R., Baker A., Scarbrough H. (2021). Professionals’ responses to the introduction of AI innovations in radiology and their implications for future adoption: A qualitative study. BMC Health Serv. Res..

[61] Arnold M. (2021). Teasing out AI in medicine: An ethical critique of AI and machine learning in medicine. J. Bioethical Inq..

[62] Coeckelbergh M. (2013). E-care as craftsmanship: Virtuous work, skilled engagement, and information technology in health care. Med. Health Care Philos..

[63] Lorenzini g., Arbelaez Ossa l., Shaw D.M., Elger B.S. (2023). Artificial intelligence and the doctor–patient relationship expanding the paradigm of shared decision making. Bioethics.

[64] Rajpurkar P., Chen E., Banerjee O., Topol E.J. (2022). AI in health and medicine. Nat. Med..

[65] Wang D., Wang L., Zhang Z., Wang D., Zhu H., Gao Y., Fan X., Tian F. “Brilliant AI doctor” in rural China: Tensions and challenges in AI-powered CDSS deployment. Proceedings of the 2021 CHI Conference on Human Factors in Computing Systems.

[66] Shortliffe E.H., Sepúlveda M.J. (2018). Clinical decision support in the era of artificial intelligence. JAMA.

[67] Cohen I.G., Graver H. (2019). A doctor’s touch: What big data in health care can teach us about predictive policing. J. Law Med. Ethics.

[68] Bauer K. (2004). Cybermedicine and the moral integrity of the physician–patient relationship. Ethics Inf. Technol..

[69] Sauerbrei A., Kerasidou A., Lucivero F., Hallowell N. (2023). The impact of artificial intelligence on the person-centred, doctor-patient relationship: Some problems and solutions. BMC Med. Inform. Decis. Mak..

[70] Salmon C., Bell K., Reyes E., Ireland E., Danek R. (2025). An analysis of telehealth in a post-pandemic rural, Midwestern community: Increased comfort and a preference for primary care. BMC Health Serv. Res..

[71] Pairon A., Philips H., Verhoeven V. (2023). A scoping review on the use and usefulness of online symptom checkers and triage systems: How to proceed?. Front. Med..

[72] Wanderås M.R., Abildsnes E., Thygesen E., Martinez S.G. (2023). Video consultation in general practice: A scoping review on use, experiences, and clinical decisions. BMC Health Serv. Res..

[73] Payne R., Clarke A., Swann N., van Dael J., Brenman N., Rosen R., Mackridge A., Moore L., Kalin A., Ladds E. (2024). Patient safety in remote primary care encounters: Multimethod qualitative study combining Safety I and Safety II analysis. BMJ Qual. Saf..

[74] Różyńska J. (2021). Taking the principle of the primacy of the human being seriously. Med. Health Care Philos..

[75] Hofmann B. (2020). The death of dignity is greatly exaggerated: Reflections 15 years after the declaration of dignity as a useless concept. Bioethics.

[76] World Medical Association WMA Statement on Augmented Intelligence in Medical Care. October 2019. https://www.wma.net/policies-post/wma-statement-on-augmented-intelligence-in-medical-care/.

[77] Hryciw B.N., Fortin Z., Ghossein J., Kyeremanteng K. (2023). Doctor-patient interactions in the age of AI: Navigating innovation and expertise. Front. Med..

[78] Bresnick J. EHR Users Want Their Time Back, and Artificial Intelligence Can Help. HealthITAnalytics, 2018. https://healthitanalytics.com/news/ehr-users-want-their-time-back-and-artificial-intelligence-can-help.

[79] Scaffardi L. (2022). La medicina alla prova dell’intelligenza artificiale. DPCE Online.

[80] Aminololama-Shakeri S., López J.E. (2019). The doctor-patient relationship with artificial intelligence. Am. J. Roentgenol..

[81] Sung J.J.Y., Savulescu J., Ngiam K.Y., An B., Ang T.L., Yeoh K.G., Cham T.J., Tsao S., Chua T.S. (2023). Artificial intelligence for gastroenterology: Singapore artificial intelligence for Gastroenterology Working Group Position Statement. J. Gastroenterol. Hepatol..

[82] Hung A.J., Chen A.B., Cacciamani G.E., Gill I.S. (2021). Artificial intelligence will (may) make doctors expendable (in good ways): Pro. Eur. Urol. Focus.

[83] Kingsford P.A., Ambrose J.A. (2024). Artificial intelligence and the doctor-patient relationship. Am. J. Med..

[84] Geantă M., Bădescu D., Chirca N., Nechita O.C., Radu C.G., Rascu S., Rădăvoi D., Sima C., Toma C., Jinga V. (2024). The Potential Impact of Large Language Models on Doctor-Patient Communication: A Case Study in Prostate Cancer. Healthcare.

[85] Zhu C. (2023). Research on Emotion Recognition-Based Smart Assistant System: Emotional Intelligence and Personalized Services. J. Syst. Manag. Sci..

[86] Finkenberg J. (2024). NASS 2023 presidential address: Artificial intelligence and its effect on the art of medicine and the physician-patient relationship. Spine J..

[87] Liu X., Keane P.A., Denniston A.K. (2018). Time to regenerate: The doctor in the age of artificial intelligence. J. R. Soc. Med..

[88] Ratkevičiūtė K., Aliukonis V. (2025). Exploring Opportunities and Challenges of AI in Primary Healthcare: A Qualitative Study with Family Doctors in Lithuania. Healthcare.

[89] Marinelli S., Basile G., Zaami S. (2022). Telemedicine, Telepsychiatry and COVID-19 Pandemic: Future Prospects for Global Health. Healthcare.

[90] Bellini V., Valente M., Gaddi A.V., Pelosi P., Bignami E. (2022). Artificial Intelligence and Telemedicine in Anesthesia: Potential and Problems. Minerva Anestesiol..

[91] Basile G., Accetta R., Marinelli S., D’Ambrosi R., Petrucci Q.A., Giorgetti A., Nuara A., Zaami S., Fozzato S. (2022). Traumatology: Adoption of the Sm@rtEven Application for the Remote Evaluation of Patients and Possible Medico-Legal Implications. J. Clin. Med..

[92] Braun M., Hummel P., Beck S., Dabrock P. (2021). Primer on an ethics of AI-based decision support systems in the clinic. J. Med. Ethics.

[93] Taddeo M., Floridi L. (2018). How AI can be a force for good. Science.

[94] Garza-Herrera R. (2024). Humans use tools: From handcrafted tools to artificial intelligence. J. Vasc. Surg. Venous Lymphat. Disord..

[95] Čartolovni A., Malešević A., Poslon L. (2023). Critical analysis of the AI impact on the patient-physician relationship: A multi-stakeholder qualitative study. Digit. Health.

[96] Geis J.R., Brady A.P., Wu C.C., Spencer J., Ranschaert E., Jaremko J.L., Langer S.G., Kitts A.B., Birch J., Shields W.F. (2019). Ethics of Artificial Intelligence in Radiology: Summary of the Joint European and North American Multisociety Statement. J. Am. Coll. Radiol..

[97] Habermas J. (1992). Moral Consciousness and Communicative Action.

[98] Jonas H. (1984). The Imperative of Responsibility: In Search of An Ethics for the Technological Age.

[99] European Parliament & Council (2016). Regulation (EU) 2016/679 of the European Parliament and of the Council of 27 April 2016 on the Protection of Natural Persons with Regard to the Processing of Personal Data and on the Free Movement of Such Data (General Data Protection Regulation, GDPR). Off. J. Eur. Union.

[100] European Parliament & Council (2024). Regulation (EU) 2024/1689 of the European Parliament and of the Council of 13 June 2024 Laying Down Harmonised Rules on Artificial Intelligence (Artificial Intelligence Act). Off. J. Eur. Union.

[101] European Parliament & Council Regulation (EU) 2017/745 of the European Parliament and of the Council of 5 April 2017 on Medical Devices, Amending Directive 2001/83/EC, Regulation (EC) No 178/2002 and Regulation (EC) No 1223/2009 and Repealing Council Directives 90/385/EEC and 93/42/EEC. https://eur-lex.europa.eu/legal-content/EN/TXT/PDF/?uri=CELEX:32017R0745.

[102] China Personal Information Protection Law, 20 August 2021. https://personalinformationprotectionlaw.com/.

[103] Han Y., Ceross A., Bergmann J. (2024). Regulatory Frameworks for AI-Enabled Medical Device Software in China: Comparative Analysis and Review of Implications for Global Manufacturer. JMIR AI.

[104] Sun S. (2024). Research on the Application of Artificial Intelligence in Medical Field from the Perspective of Behavioral Law. Beijing Law Rev..

[105] Food and Drug Administration Clinical Decision Support Software. Guidance for Industry and Food and Drug Administration Staff, 28 September 2022. https://www.fda.gov/media/109618/download?utm_source=chatgpt.com.

[106] Food and Drug Administration, Artificial Intelligence/Machine Learning (AI/ML)-Based Software as a Medical Device (SaMD) Action Plan, January 2021. https://www.fda.gov/media/145022/download?utm_source=chatgpt.com.

[107] AMA, Augmented Intelligence Development, Deployment, and Use in Health Care, November 2024; p. 16. https://www.ama-assn.org/system/files/ama-ai-principles.pdf?utm_source=chatgpt.com.

[108] Zaami S., Melcarne R., Patrone R., Gullo G., Negro F., Napoletano G., Monti M., Aceti V., Panarese A., Borcea M.C. (2022). Oncofertility and Reproductive Counseling in Patients with Breast Cancer: A Retrospective Study. J. Clin. Med..

[109] Zampatti S., Peconi C., Megalizzi D., Calvino G., Trastulli G., Cascella R., Strafella C., Caltagirone C., Giardina E. (2024). Innovations in Medicine: Exploring ChatGPT’s Impact on Rare Disorder Management. Genes.

[110] Gulìa C., Signore F., Gaffi M., Gigli S., Votino R., Nucciotti R., Bertacca L., Zaami S., Baffa A., Santini E. (2020). Y RNA: An Overview of Their Role as Potential Biomarkers and Molecular Targets in Human Cancers. Cancers.

[111] Meekins-Doherty L., Dive L., McEwen A., Sexton A. (2025). Generative AI and the Profession of Genetic Counseling. J. Genet. Couns..

[112] Piergentili R., Del Rio A., Signore F., Umani Ronchi F., Marinelli E., Zaami S. (2021). CRISPR-Cas and Its Wide-Ranging Applications: From Human Genome Editing to Environmental Implications, Technical Limitations, Hazards and Bioethical Issues. Cells.

[113] Johnson K.B., Wei W.-Q., Weeraratne D., Frisse M.E., Misulis K., Rhee K., Zhao J., Snowdon J.L. (2021). Precision Medicine, AI, and the Future of Personalized Health Care. Clin. Transl. Sci..

[114] Mello M.M., Char D., Xu S.H. (2025). Ethical Obligations to Inform Patients About Use of AI Tools. JAMA.

[115] Devadas R.M., Sowmya T. (2025). Quantum Machine Learning: A Comprehensive Review of Integrating AI with Quantum Computing for Computational Advancements. MethodsX.

[116] Durant T.J.S., Knight E., Nelson B., Dudgeon S., Lee S.J., Walliman D., Young H.P., Ohno-Machado L., Schulz W.L. (2024). A Primer for Quantum Computing and Its Applications to Healthcare and Biomedical Research. J. Am. Med. Inform. Assoc..

